# EnACP: An Ensemble Learning Model for Identification of Anticancer Peptides

**DOI:** 10.3389/fgene.2020.00760

**Published:** 2020-07-30

**Authors:** Ruiquan Ge, Guanwen Feng, Xiaoyang Jing, Renfeng Zhang, Pu Wang, Qing Wu

**Affiliations:** ^1^Key Laboratory of Complex Systems Modeling and Simulation, School of Computer Science and Technology, Hangzhou Dianzi University, Hangzhou, China; ^2^Xi'an Key Laboratory of Big Data and Intelligent Vision, School of Computer Science and Technology, Xidian University, Xi'an, China; ^3^Toyota Technological Institute at Chicago, Chicago, IL, United States; ^4^Shandong Provincial Hospital Affiliated to Shandong First Medical University, Jinan, China; ^5^Computer School, Hubei University of Arts and Science, Xiangyang, China

**Keywords:** anticancer peptides, feature representation, ensemble learning, pseudo amino acid composition, system biology

## Abstract

As cancer remains one of the main threats of human life, developing efficient cancer treatments is urgent. Anticancer peptides, which could overcome the significant side effects and poor results of traditional cancer treatments, have become a new potential alternative these years. However, identifying anticancer peptides by experimental methods is time consuming and resource consuming, it is of great significance to develop effective computational tools to quickly and accurately identify potential anticancer peptides from amino acid sequences. For most current computational methods, feature representation plays a key role in their final successes. This study proposes a novel fast and accurate approach to identify anticancer peptides using diversified feature representations and ensemble learning method. For the feature representations, the information is encoded from multidimensional feature spaces, including sequence composition, sequence-order, physicochemical properties, etc. In order to better model the potential relationships of peptides, multiple ensemble classifiers, LightGBMs, are applied to detect the different feature sets at first. Then the obtained multiple outputs are used as inputs of the support vector machine classifier, which effectively identifies anticancer peptides. Experimental results on cross validation and independent test sets demonstrate that our method can achieve better or comparable performances compared with other state-of-the-art methods.

## Introduction

Cancer has become a common disease in humans, and it often leads to a higher mortality rate, especially in developing and developed countries (Ortega-Garcia et al., 2020). The complexity and heterogeneity of cancer are major obstacles for anticancer therapy development (Kasak and Laan, 2020; Umbreit et al., 2020). Traditional cancer treatments, such as radiation therapy, targeted therapy and chemotherapy, often fail to distinguish cancer cells from normal cells. Traditional surgery could not guarantee the precise removal of the diseased part, which is seriously harmful to the patient's body (An et al., 2019). At the same time, the risk of recurrence after surgery is high. In addition, cancer cells have developed resistance to traditional anticancer drugs due to their overuse. Overall, traditional treatment methods have obvious side effects and poor results. In view of these problems, there is an urgent to discover and design novel cancer treatments and anticancer agents to fight against this deadly disease (Esfandiari Mazandaran et al., 2019; Sima et al., 2019; Bahuguna et al., 2020).

In recent years, peptide-based therapy has become a potential method of cancer treatments. This method can target and kill cancer cells while do not impair the normal cells (Harris, 2020). Anticancer peptides (ACPs) with short amino acid sequences can avoid the disadvantages of traditional cancer treatments. They generally have the characteristics of high specificity, high tissue penetration, low production cost, toxic under normal physiological functions, ease of synthesis and modification, etc. And natural ACPs are safer than synthetic drugs (Feng and Wang, 2019). The electrostatic interactions between ACPs and cancer cell membranes are considered to be one of the main factors for the selective killing of cancer cells (Lin et al., 2018; Naguib et al., 2018). They are believed to play a vital role in the selective toxicity of ACPs to cancer. Currently, many approved peptide-based drugs are being evaluated in various stages of clinical trials (Tesauro et al., 2019; Brunetti et al., 2020). As more and more ACPs are identified and verified by experiments, it is found that most ACPs are derived from protein sequences (Tyagi et al., 2013). However, the discovery of novel ACPs from wet-lab experimentation is laborious, time-consuming and expensive. So, it is essential to develop efficient computational methods to rapidly identify potential ACPs from the peptide sequences.

In the past decade, the accurate identification of ACPs from peptide sequences remains an open research topic in the field of bioinformatics and immunoinformatic. Machine learning methods have been widely used to identify ACPs in many researches. It mainly includes two key techniques which are feature representation and classifier. For feature representation, if the features of peptide sequences are well-extracted, it will be easier to precisely predict the ACPs (Jing et al., 2019). At present, some tools in the prediction of ACPs have been developed. The first computational tool is called Anti-CP (Tyagi et al., 2013), which encoded peptides with sequence-based features and binary profiles to predict ACPs based on Support Vector Machine (SVM). In another work, Hajisharifi et al. considered two kinds features from the local correlation and Chou's pseudo acid amino composition (PseAAC) to improve the prediction of ACPs (Hajisharifi et al., 2014). ACPP used an improved feature encoding method via three type of protein relatedness measure, integrating compositional information, centroidal and distributional information of amino acids (Vijayakumar and Lakshmi, 2015). iACP has referred that membrane interactions are related to their conformation or the order of amino acids. And, it can get better results through cross validation and optimizing the g-gap dipeptide components method compared to the previous predictors (Chen W. et al., 2016). Li et al. indicated that the different types of feature combinations can improve the prediction for ACPs (Chen W. et al., 2016). MLACP constructed features using amino acid composition, atomic composition, dipeptide composition, and physicochemical properties and developed SVM and random forest (RF) methods to predict ACPs (Manavalan et al., 2017). SAP employed 400D features with g-gap dipeptide information and feature selection to identify ACPs (Xu et al., 2018). ACPred-FL can orderly extract effective features from sequence-based feature and a group of SVM models (Wei et al., 2018). mACPpred explored seven feature encodings and a two-step feature selection method to exclude irrelevant features (Ge et al., 2016; Boopathi et al., 2019). Then, the obtained features are input into SVM classifier to gain the predicted result. In addition, a special repository named CancerPPD was collected and created with the manually verified ACPs from the published literature, patents and other databases (Tyagi et al., 2015). It provides a wealth of information related to the peptide for research and experimental personnel to use for reference such as its origin, the nature of the peptide, anticancer activity, terminal modification, conformation, etc. The information is helpful to understand the comprehensive properties of ACPs. And it also provides a reference for the design and identification of ACPs (Lin et al., 2015).

In this paper, we propose a novel two-step prediction model EnACP to accurately identify the ACPs. At first, feature representation is composed of four categories: amino acid composition, autocorrelation, pseudo amino acid composition and profile-based features (Chen et al., 2018). Each type includes a few modes. Finally, 19 kinds of feature patterns are generated. For each feature pattern, LightGBM (Light Gradient Boosting Machine) classifier is employed to generate the initial prediction (Ke et al., 2017). The former predicted results as the new features are input to SVM classifier to get the final prediction. Cross validation results showed that the proposed EnACP model performed better than the previous methods. Furthermore, EnACP achieved comparable performances compared with the existing methods on a new independent dataset. EnACP is available at https://github.com/greyspring/EnACP.

## Materials and Methods

### Dataset

In this study, we use two groups of ACP datasets from the existed literatures to evaluate the performance of the proposed method. For them, one dataset is used to test the cross-validation performance compared with the existing models (Hajisharifi et al., 2014). The other with an independent test dataset can better measure the generalization capability of the model (Boopathi et al., 2019).

**Figure 1:**
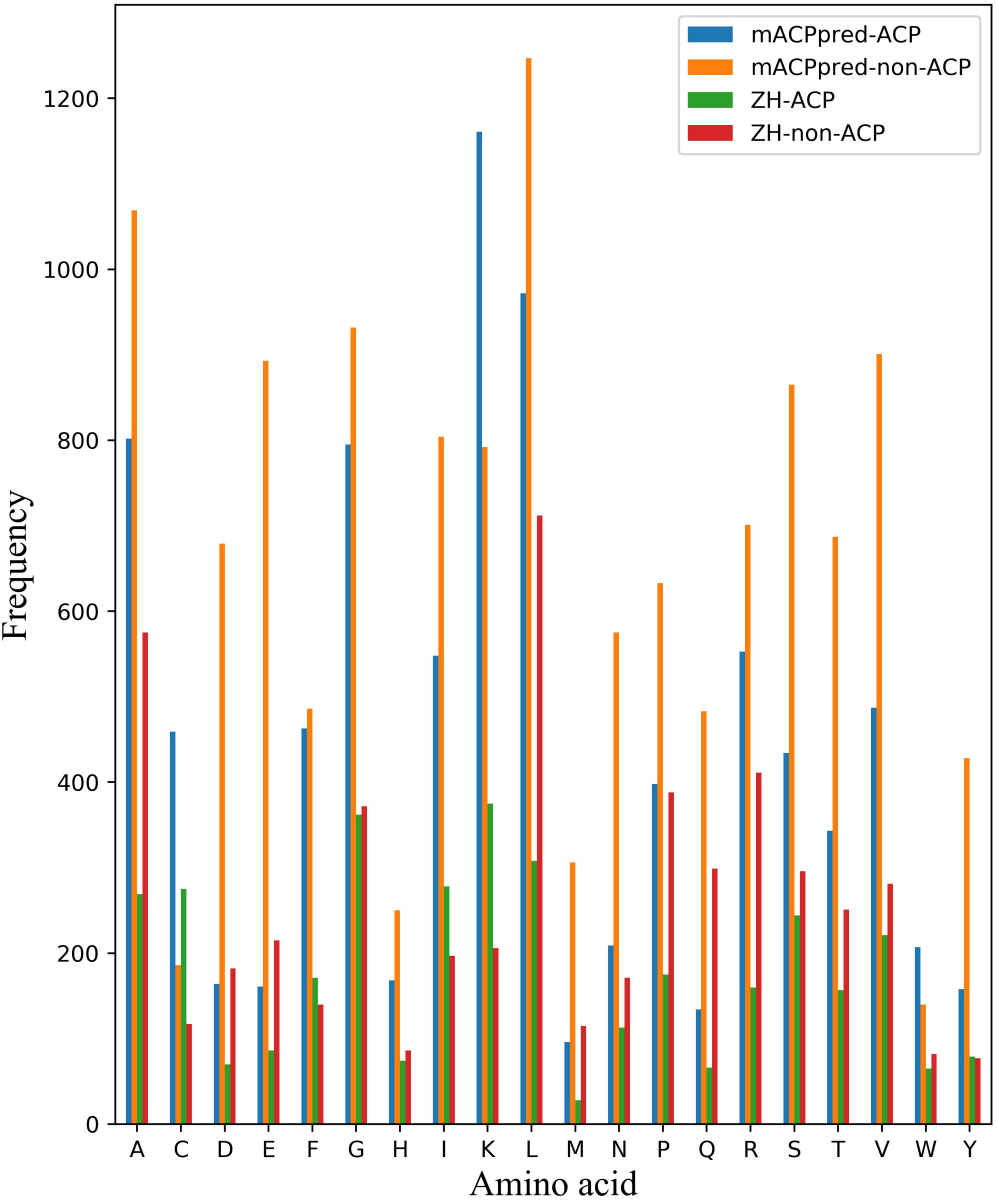
Amino acid frequency distribution on cross-validation and independent datasets. The number of 20 amino acids are counted in mACPpred and ZH datasets. The horizontal axis represents the abbreviation of 20 amino acids. The ordinate represents the number of amino acids.

**Figure 2:**
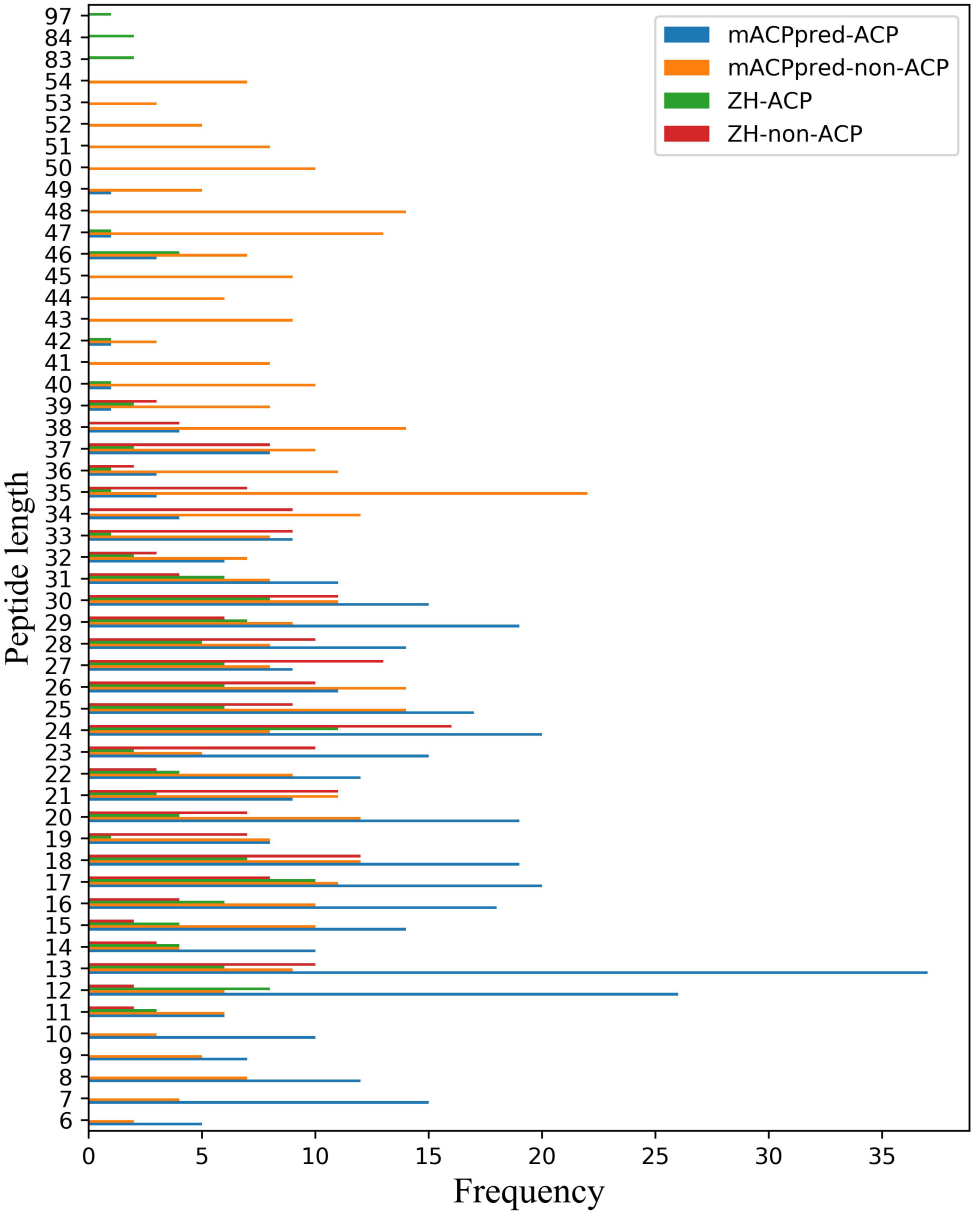
Peptide length distribution of ACP and non-ACP on mACPpred and ZH datasets. The horizontal axis represents the number of statistics. The ordinate represents the length of the peptide sequence.

For the two datasets, one is called ZH dataset including 138 ACPs and 206 non-ACPs for the 5-fold cross-validation test. The other is from mACPpred for the independent test. In mACPpred dataset, the training dataset consists of 266 ACPs and 266 non-ACPs, and the independent dataset consists of 157 ACPs and 157 non-ACPs. The two group datasets have the low redundancy which were processed to prevent homology bias and high similarity in the related literatures. Amino acid frequency distribution of ACP and non-ACP in the two datasets are shown in Figure 1. The sequences containing not 20 natural amino acids are eliminated. From Figure 2, most of the peptide sequences are between 5 and 50 in length in the two datasets especially in mACPpred-ACP and ZH-non-ACP. For mACPpred-non-ACP and ZH-ACP, their ratio is 94.6 and 96.4%, respectively.

### Features Representation

There are 19 kinds of features in total used in this study, three of which belong to amino acid composition, four of which belong to autocorrelation features, four of which belong to pseudo amino acid composition, and eight of which belong to profile-based features (Liu et al., 2015, 2017; Liu, 2019).

#### Amino Acid Composition

Basic kmer (Kmer) (Liu et al., 2008) is a very simple feature extraction method that represents any peptide sequence as a vector consisted of occurrence frequencies of k neighboring amino acids. Distance-based Residue (DR) (Liu et al., 2014b) extracts features from sequence by counting the occurrence frequencies of all possible residue pairs within a certain distance. Just like the DR method, the method of Distance-Pairs and reduced alphabet scheme (Distance Pair) (Liu et al., 2014a) also extracts features from sequence by counting the occurrence frequencies of residue pairs within a certain distance, except that the residue types are reduced by clustering.

#### Autocorrelation Features

A peptide sequence P is often formulated in the following format, with the N-terminus at the left, and the C-terminus at the right.

$$\begin{array}{l} \text{P}={\text{R}}_{1}{\text{R}}_{2}{\text{R}}_{3}\cdot \cdot \cdot {\text{R}}_{\text{L}} \end{array}$$

where R_1_ represents the 1st amino acid, R_2_ represents the 2nd amino acid, and so forth.

Given a physicochemical index of amino acids, The Auto covariance (AC) (Cao et al., 2013) approach measures the correlation between two residues separated by distance d, which can be calculated as:

$$\begin{array}{l} \text{AC}\left(u,d\right)=\overset{L-d}{\underset{i=1}{\sum }}\left(I_{u}\left(R_{i}\right)-{\bar{I}}_{u}\right)\left(I_{u}\left(R_{i+d}\right)-{\bar{I}}_{u}\right)/\left(L-d\right) \end{array}$$

where *u* indicates the physicochemical index, *I*_*u*_(*R*_*i*_) means the index value of *R*_*i*_, and ${\bar{I}}_{u}$ is the average index value along the whole sequence:

$$\begin{array}{l} {\bar{I}}_{u}=\overset{L}{\underset{i=1}{\sum }}I_{u}\left(R_{i}\right)/L \end{array}$$

The Cross covariance (CC) (Cao et al., 2013) approach measures the correlation between two residues separated by distance d based on two different physicochemical indices, which can be calculated by:

$$\begin{array}{l} \text{CC}\left(u,v,d\right)=\overset{L-d}{\underset{i=1}{\sum }}\left(I_{u}\left(R_{i}\right)-{\bar{I}}_{u}\right)\left(I_{v}\left(R_{i+d}\right)-{\bar{I}}_{v}\right)/\left(L-d\right) \end{array}$$

where *u* and *v* indicate two different indices, *I*_*u*_(*R*_*i*_)(*I*_*v*_(*R*_*i*_)) means the index value of *R*_*i*_, and ${\bar{I}}_{u}$(${\bar{I}}_{v}$) is the average index value along the whole sequence.

Auto-cross covariance (ACC) (Cao et al., 2013) is the combination of AC and CC. Physicochemical distance transformation (PDT) (Liu et al., 2012) is a sequence-based method, in which any peptide sequence is firstly encoded as a series of numbers by amino acid index (AAindex) (Kawashima et al., 2008), and then a fixed length vector is extracted through distance transformation.

#### Pseudo Amino Acid Composition

Parallel correlation pseudo amino acid composition (PC-PseAAC) (Chou, 2001) is an approach that takes the sequence-order information into account and represents any peptide sequence as:

$$\begin{array}{l} \text{P}=\left[x_{1}\;x_{2}\;x_{3}\;\cdots \;x_{20}\;x_{20+1}\;\cdots \;x_{20+\lambda }\right] \end{array}$$

where

$$\begin{array}{l} x_{u}=\{\begin{array}{l} \frac{f_{u}}{\overset{20}{\underset{i=1}{\sum }}f_{i}+w\overset{\lambda }{\underset{j=1}{\sum }}\theta_{j}}\;(1\leq u\leq 20)\\ \frac{w\theta_{u-20}}{\overset{20}{\underset{i=1}{\sum }}f_{i}+w\overset{\lambda }{\underset{j=1}{\sum }}\theta_{j}}\;(20+1\leq u\leq 20+\lambda ) \end{array} \end{array}$$

where *f*_*i*_(*i* = 1,2,…,20) is the occurrence frequency of the 20 native amino acids in the peptide; the integer λ represents the highest tier of correlation along the sequence; *w* is the weight factor ranging from 0 to 1; θ_*j*_(*j*=1, 2, …, λ) is the *j*-tier correlation factor that is defined as:

$$\begin{array}{l} \theta_{j}=\frac{1}{L-j}\overset{L-j}{\underset{i=1}{\sum }}\Theta \left(R_{i},R_{i+j}\right)\;(1\leq j\leq \lambda ) \end{array}$$

Where the correlation function is given by

$$\begin{array}{l} \Theta \left(R_{i},R_{j}\right)=\frac{1}{3}\{{\left[H_{1}(R_{i})-H_{1}(R_{j})\right]}^{2}+{\left[H_{2}(R_{i})-H_{2}(R_{j})\right]}^{2}\\ \;+{\left[M(R_{i})-M(R_{j})\right]}^{2}\} \end{array}$$

where *H*^1^(*R*_*i*_), *H*^2^(*R*_*i*_), and *M*(*R*_*i*_) are the standardized hydrophobicity value, hydrophilicity value, and side-chain mass of *R*_*i*_, respectively.

Series correlation pseudo amino acid composition (SC-PseAAC) (Chou, 2005) is a variant of PC-PseAAC that represents any peptide sequence as:

$$\begin{array}{l} \text{P}=\;\left[x_{1}\;\cdots \;x_{20}\;x_{20+1}\;\cdots \;x_{20+\lambda }\;x_{20+\lambda +1}\;\cdots \;x_{20+2\lambda }\right] \end{array}$$

where

$$\begin{array}{l} x_{u}=\{\begin{array}{l} \frac{f_{u}}{\overset{20}{\underset{i=1}{\sum }}f_{i}+w\overset{2\lambda }{\underset{j=1}{\sum }}\theta_{j}}\;\left(1\leq u\leq 20\right)\\ \frac{w\theta_{u-20}}{\overset{20}{\underset{i=1}{\sum }}f_{i}+w\overset{2\lambda }{\underset{j=1}{\sum }}\theta_{j}}\;\left(20+1\leq u\leq 20+2\lambda \right) \end{array} \end{array}$$

where *f*_*i*_(*i*=1,2,…,20) is the occurrence frequency of the 20 native amino acids in the peptide; the integer λ represents the highest tier of correlation along the sequence; *w* is the weight factor ranging from 0 to 1; θ_*j*_(*j*=1, 2, …, 2λ) is the *j*-tier correlation factor that is defined as:

$$\begin{array}{l} \{\begin{array}{l} \theta_{1}=\frac{1}{L-1}\overset{L-1}{\underset{i=1}{\sum }}H_{i,i+1}^{1}\\ \theta_{2}=\frac{1}{L-1}\overset{L-1}{\underset{i=1}{\sum }}H_{i,i+1}^{2}\\ \cdots \\ \theta_{2\lambda -1}=\frac{1}{L-\lambda }\overset{L-\lambda }{\underset{i=1}{\sum }}H_{i,i+\lambda }^{1}\\ \theta_{2\lambda }=\frac{1}{L-\lambda }\overset{L-\lambda }{\underset{i=1}{\sum }}H_{i,i+\lambda }^{2} \end{array} \end{array}$$

where the correlation functions are given by

$$\begin{array}{l} \{\begin{array}{c} H_{i,j}^{1}=h^{1}\left(R_{i}\right)\cdot h^{1}\left(R_{j}\right)\\ H_{i,j}^{2}=h^{2}\left(R_{i}\right)\cdot h^{2}\left(R_{j}\right) \end{array} \end{array}$$

where *h*^1^(*R*_*i*_) and *h*^2^(*R*_*i*_) are the standardized hydrophobicity and hydrophilicity values of *R*_*i*_, respectively.

General parallel correlation pseudo amino acid composition (PC-PseAAC-General) is an enhanced version of PC-PseAAC, in which both the built-in indices extracted from AAindex and the indices provided by users can be incorporated. General series correlation pseudo amino acid composition (SC-PseAAC-General) is an enhanced version of SC-PseAAC, in which both the built-in indices extracted from AAindex and the indices provided by users can be incorporated.

#### Profile-Based Features

The Top-n-gram (Liu et al., 2014b) approach extracts evolutionary information from the frequency profiles calculated from the multiple sequence alignments outputted by PSI-BLAST (Altschul et al., 1997), and any peptide sequence is represented as a fixed dimension feature vector by counting the occurrence times of each Top-n-gram. Profile-based physicochemical distance transformation (PDT-Profile) is similar with PDT except that the features are extracted from frequency profiles. Distance-based Top-n-gram (DT) extends the original Top-n-gram approach by considering the relative position information of Top-n-gram pairs in peptide sequences, and the feature vector of peptide sequence was calculated by counting the occurrences of all possible Top-n-gram pairs within a certain distance threshold.

Profile-based Auto covariance (AC-PSSM) (Dong et al., 2009) transforms the PSSM of a peptide into fixed-length vector, in which the AC variable measures the correlation of the same property between two residues separated by a distance. Profile-based Cross covariance (CC-PSSM) (Dong et al., 2009) transforms the PSSM of a peptide into fixed-length vector, in which the CC variables measure the correlation of two different properties between two residues separated by a distance. Profile-based Auto-cross covariance (ACC-PSSM) (Dong et al., 2009) represents any peptide sequence as a feature vector consisting of ACC variables that are the combination of AC variables and CC variables. PSSM distance transformation (PSSM-DT) (Xu et al., 2015) extracts features from the PSSM of a peptide which measure the occurrence probabilities of any amino acid pairs separated by a distance. PSSM relation transformation (PSSM-RT) (Zhou et al., 2017) extracts features from the PSSM of a peptide by utilizing the relationships of evolutionary information between residues.

### Support Vector Machine and LightGBM

In this study, the dataset has exactly two class labels: anticancer peptides (positive) and non-anticancer peptides (negative). Support vector machines (SVMs) are very suitable for binary classification, and because of the strong generalization ability for small datasets, they are used extensively in biomedical data mining (Chen et al., 2019; Jiang et al., 2020). SVM classifies data by finding the best hyperplane to separate all data points of one class from these of another class. The best hyperplane of SVM is the hyperplane with the largest margin between two classes. SVM is firstly proposed for linearly separable data, and when the data are non-separable, the kernel functions such as radial basis function can be used.

LightGBM (Light Gradient Boosting Machine) is a distributed gradient lifting framework based on decision tree algorithm proposed by Microsoft in 2017 (Ke et al., 2017). In order to shorten the computation time, LightGBM as a good ensemble learning algorithm was designed for two main reasons (Xia et al., 2017). For one thing, it can reduce the use of memory and the communication cost, improves the efficiency when multiple machines are parallel. For another thing, it designs and implements a good strategy for feature selection.

### Methodology

**Figure 3:**
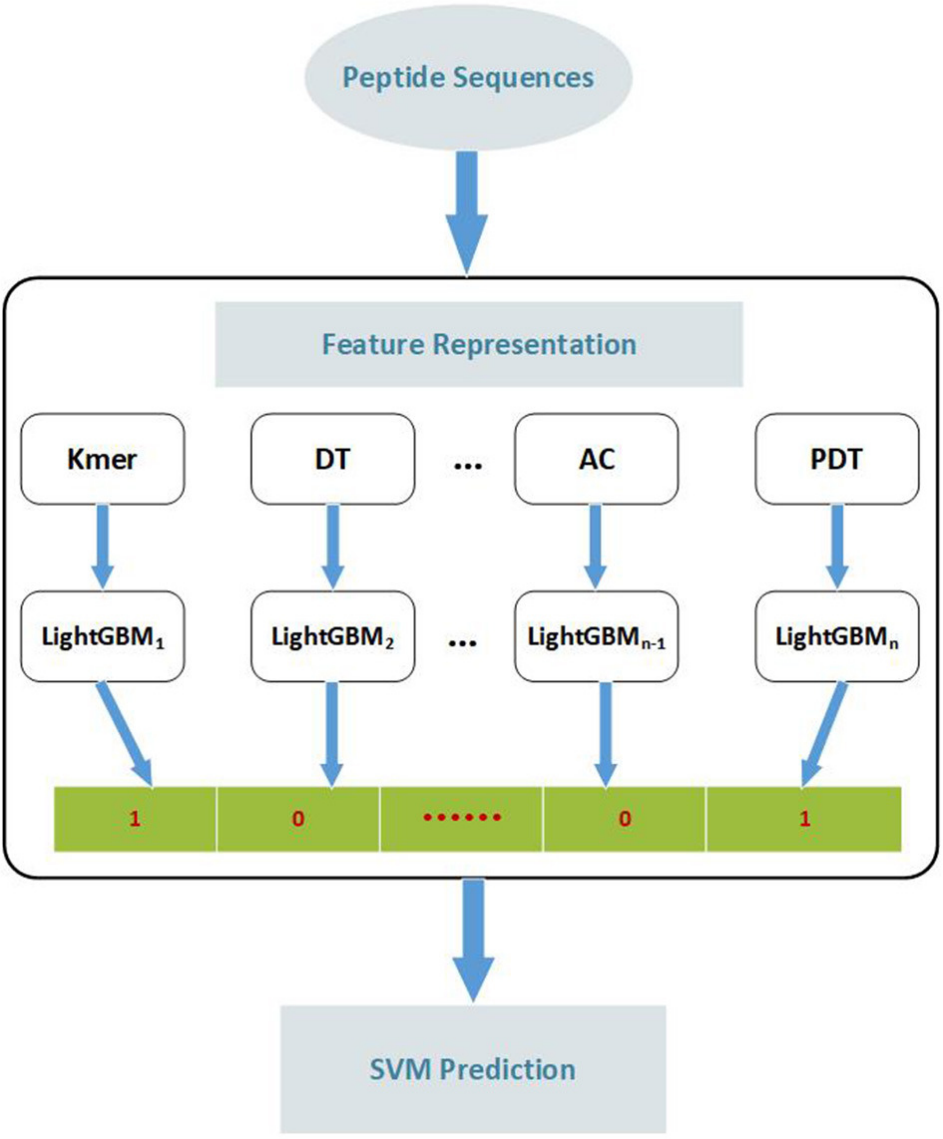
The flow diagram of identifying anticancer peptides. Peptide sequences are represented by 19 feature extraction methods. According to the features obtained by each extraction method, LightGBM is employed to classify the peptide sequences. Then, the outputs of all LightGBMs are input into SVM classifier to predict the peptide sequence as ACP or non-ACP. Kmer: subsequences of length K contained within a peptide sequence; DT, Distance-based Top-n-gram; AC, Auto Covariance; PDT, Physicochemical Distance Transformation; SVM, Support Vector Machine.

To develop an accurate predictor of ACPs, we present a two-step ensemble learning method called EnACP. The framework of the model is shown in Figure 3. In the first step, 19 feature encodings of the peptide sequences are extracted in terms of amino acid composition, autocorrelation, pseudo amino acid composition and profile-based features as descripted in section Features Representation. For each group of feature encodings, the initial prediction is obtained separately using an ensemble learning classifier LightGBM. In this way, the complex higher-dimensional features are dispersed to lower dimensions. Then, the outputs of all LightGBMs as combinative nineteen-dimensional feature vector are input into an optimized SVM classifier to capture the hidden relationships. At last, the peptide sequence is identified whether it is ACP or non-ACP.

For a given binary classification problem about a set of sequences *Q(s)*, the class labels *C*={*C*_1_, *C*_2_, …, *C*_*s*_}, *C*_*i*_ϵ{*0, 1*}, and each sample *q*_*i*_ has *k* group features < *F*_1_(*q*_*i*_), *F*_2_(*q*_*i*_),. *F*_*k*_(*q*_*i*_) > , where *F*_*j*_ is the *j*^*th*^ group features. Each group has several related features. Firstly, all the features are generated by the 19 kinds of feature representation algorithm for all the sequences. For the train dataset, LightGBM is employed to classify each group features, respectively. The LightGBM classification results of k group features are input SVM to train the model. For the test dataset, the inputs are generated according to the first layer model of the train data set. Finally, ACPs or non-ACPs are identified for the test peptide sequences. The algorithm flow is described in the following pseudocode.

As shown from the pseudocode, there are three factors that affect the time complexity of the model EnACP, such as feature extraction, LightGBM and SVM algorithms. Let *p* and *n* be the numbers of the most features *F*_*i*_*(q*_*i*_*)* and train samples *Q*_*t*_, respectively. And the length of the longest sequence is *l*. Different feature extraction methods are relatively independent, and they can be generated in parallel. So, the most complex feature extraction method determines the time complexity of the feature extraction stage. For the 19 groups of feature extraction methods, the profile-based method with the highest complexity is O(n^*^l^3^). LightGBM is implemented using three technologies to improve the model efficiency: gradient-based one-side sampling, exclusive feature bundling, and histogram algorithm. These techniques have resulted in more or less a reduction in the number of samples and features. Moreover, it also supports feature parallel and data parallel processing. So, its worst time complexity will not exceed O(p^*^ *n*). And the computational complexity of an SVM is O(*n*^3^) for the training dataset. So the worst-case time complexity of EnACP is max(O(n^*^l^3^), O(p^*^n), O(*n*^3^)-). But most of the features will usually be excluded in the first layer. Then the SVM algorithm in the second layer will be significantly speeded up. So the actual calculation time will not reach the upper-bound in the train stage. For the test dataset, the time is mainly consumed in the feature extraction stage after the parameters of LightGBM and SVM are optimized.

**Algorithm: *EnACP***

**Input:** a sequences set *Q: (q*_*i*_*,C*_*i*_*), k* groups of feature types, class label *C*_*i*_={*0, 1*}, *q*_*i*_ is a peptide sequence*, Q*_*t*_ *is train dataset, Q*_*v*_ *is test dataset*.

**Begin**

1. **for** each sequence *q*_*i*_ in *Q*:

        *// Initialize all features of q*_*i*_*, each F*_*i*_*(q*_*i*_*) represent one group of features*

       2. *F(q*_*i*_*)* = < *F*_1_*(q*_*i*_*), F*_2_*(q*_*i*_*),., F*_*k*_*(q*_*i*_*)* > = *{}*

        // Initialize second level features

        3. *L2FK(q*_*i*_*)[1.k]* =*{}*

        // Feature extract

        4. **for** *j* = 1 to *k*

           5. Generate features *F*_*j*_*(q*_*i*_*)* according to feature representation algorithm *F*_*j*_

        6. **endfor**

7. **endfor**

8. **for** train dataset *Q*_*t*_: *(q*_*t*_*,C*_*t*_*)*

        9. **for** *m* = 1 to *k*

*                // Classify the sequences Q*_*t*_ *in the first level*

           10. *L1Model*_*m*_= *LightGBM(F(q*_*t*_*), C*_*t*_*)*

           11. *L2FK(q*_*t*_*)[m]*= *L1Model*_*m*_*(F(q*_*t*_*)-)*

        12. **endfor**

                // Train the model in the second level

        13. *L2Model*=*SVM (L2FK(q*_*t*_*) [1.k], C*_*t*_*)*

14. **endfor**

15. **for** test dataset *Q*_*v*_*: (q*_*v*_*,C*_*v*_*)*

        16. **for** *n* = 1 to *k*

                // Classify the sequences Qv in the first level

              17. *L2FK(q*_*v*_*) [n]*= *L1Modeln(F(q*_*v*_*)-)*

        18. **endfor**

*                // Predict the peptide sequence q*_*v*_*: ACP or non-ACP*

        19. *FinalPredict(q*_*v*_*)*= *L2Model(L2FK(q*_*v*_*) [1.k])*

20. **endfor**

**End**

### Evaluation

The metrics for performance evaluation used in our experiments include Receiver Operating Characteristic curve (ROC), Area Under a ROC Curve (AUC), Sensitivity (Sn), Specificity (Sp), Accuracy (Acc), and the Matthews correlation coefficient (MCC) (Plyusnin et al., 2019). Suppose TP, FP, TN and FN are the abbreviations for true positives, false positives, true negatives, and false negatives respectively, then the evaluation metrics can be calculated as:

$$\begin{array}{l} \;Sp=\frac{TN}{TN+FP}\\ \;Sn=\frac{TP}{TP+FN}\\ \;Acc=\frac{TP+TN}{TP+TN+FP+FN}\\ MCC=\frac{TP\cdot TN-FP\cdot FN}{\sqrt{\left(TP+FP\right)\cdot \left(TP+FN\right)\cdot \left(TN+FP\right)\cdot \left(TN+FN\right)}} \end{array}$$

## Results

### Performance on Different Feature Representations

**Figure 4:**
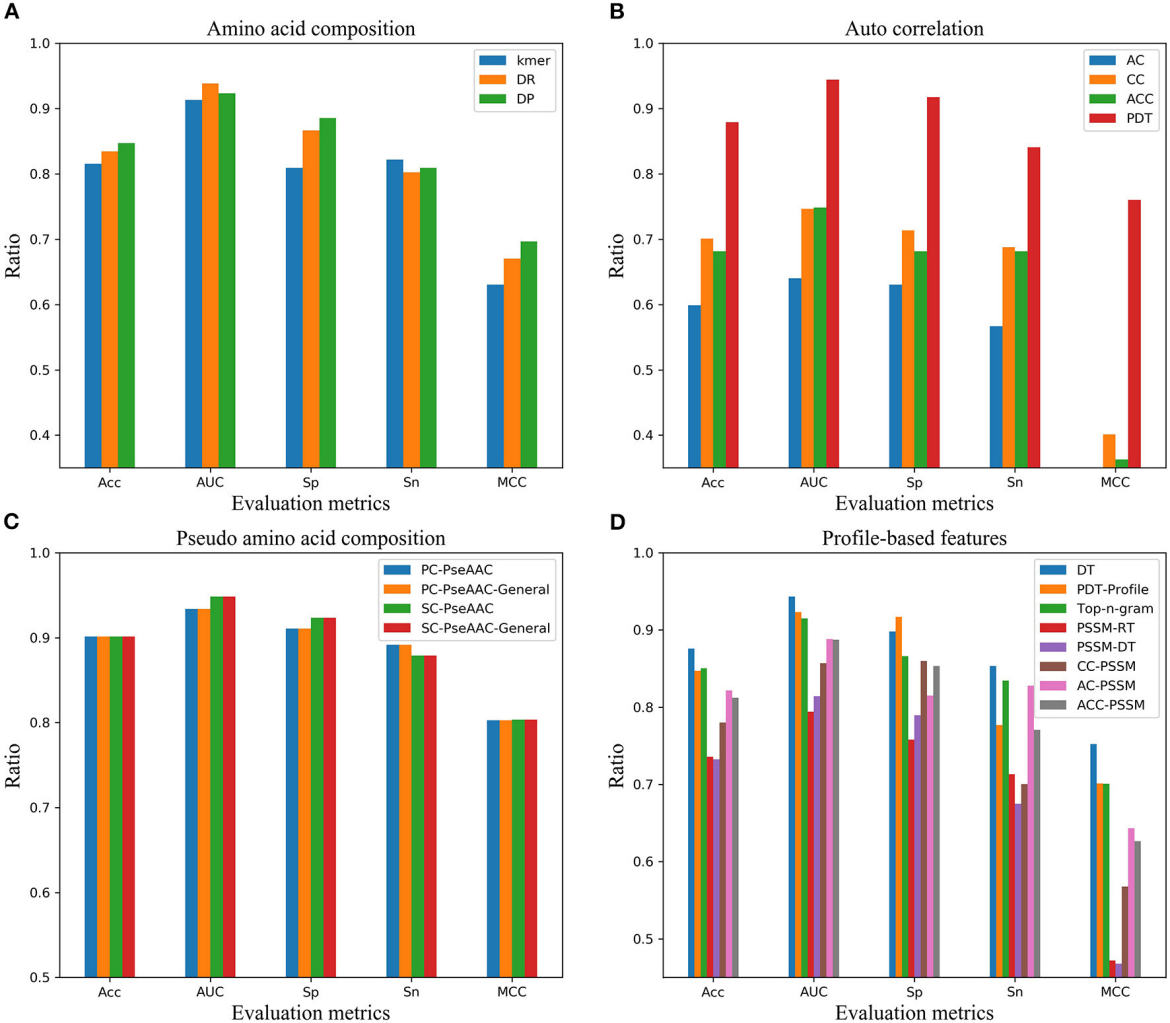
Performance of four kinds of feature encodings on independent dataset in AUC, Sn, Sp, Acc, and MCC. **(A)** Performance of feature encodings based on amino acid composition**, (B)** Performance of feature encodings based on autocorrelation**, (C)** Performance of feature encodings based on pseudo amino acid composition, **(D)** Performance of feature encodings based on profile.

In order to find the effective feature coding representation of the peptide sequence, four kinds of feature representation methods including 19 feature encodings were extracted in terms of amino acid composition, autocorrelation, pseudo amino acid composition and profile-based features. Referring to the first step of the model, the ACPs were identified by LightGBM classifier using various feature codes, respectively. From the overall results in Figure 4, they were ranked by pseudo amino acid composition, amino acid composition, profile-based features and autocorrelation. In terms of the various feature codes, pseudo amino acid composition worked best according to the value of the performance indexes Acc, AUC, Sp, Sn, and MCC. Its MCC was nearly 14 percentage points higher than the second place. And, its Acc, Sn, and Sp were about 7 percentage points higher than the second-place method from amino acid composition. Among them, autocorrelation encoding was the worst, and its performance indexes were all below 80%.

### Performance Comparison on Cross-Validation Dataset

To verify the effect of our model, we compared the results of a few popular methods such as Li method (Li and Wang, 2016), ZH method (Hajisharifi et al., 2014) and iACP (Chen W. et al., 2016) on ZH dataset with 5-fold cross-validation. In order to compare the predictive capability, the predicted results of the four methods were showed in Table 1. Judging from the result, our predictor EnACP performed better than other three methods and reached the first place in the evaluation indexes on Sn, Acc, and MCC. In all the evaluation indexes, EnACP only lost to iACP in Sp index. Acc, Sn, and MCC of our method were about 0.6 to 5.7%, 2.2 to 7.6%, and 1.7 to 12.6% higher than the predictive results of other methods, respectively. In terms of Sp index, our method was only 0.9% lower than iACP method, but also much higher than other methods. From the discussion above, it can be seen that our method may automatically learn representative features from the numerous feature codes. The two step combined classifiers with LightGBM and SVM may improve the accuracy of prediction and achieve better identification efficiency between ACPs and non-ACPs.

**Table 1:** Performance comparison of different methods on 5-fold cross-validation dataset.

**Methods**	**Acc**	**Sn**	**Sp**	**MCC**
EnACP	0.954	0.928	0.981	0.910
Li method	0.942	0.906	0.967	0.879
ZH method	0.897	0.852	0.927	0.784
iACP	0.948	0.884	0.990	0.893

**Figure 5:**
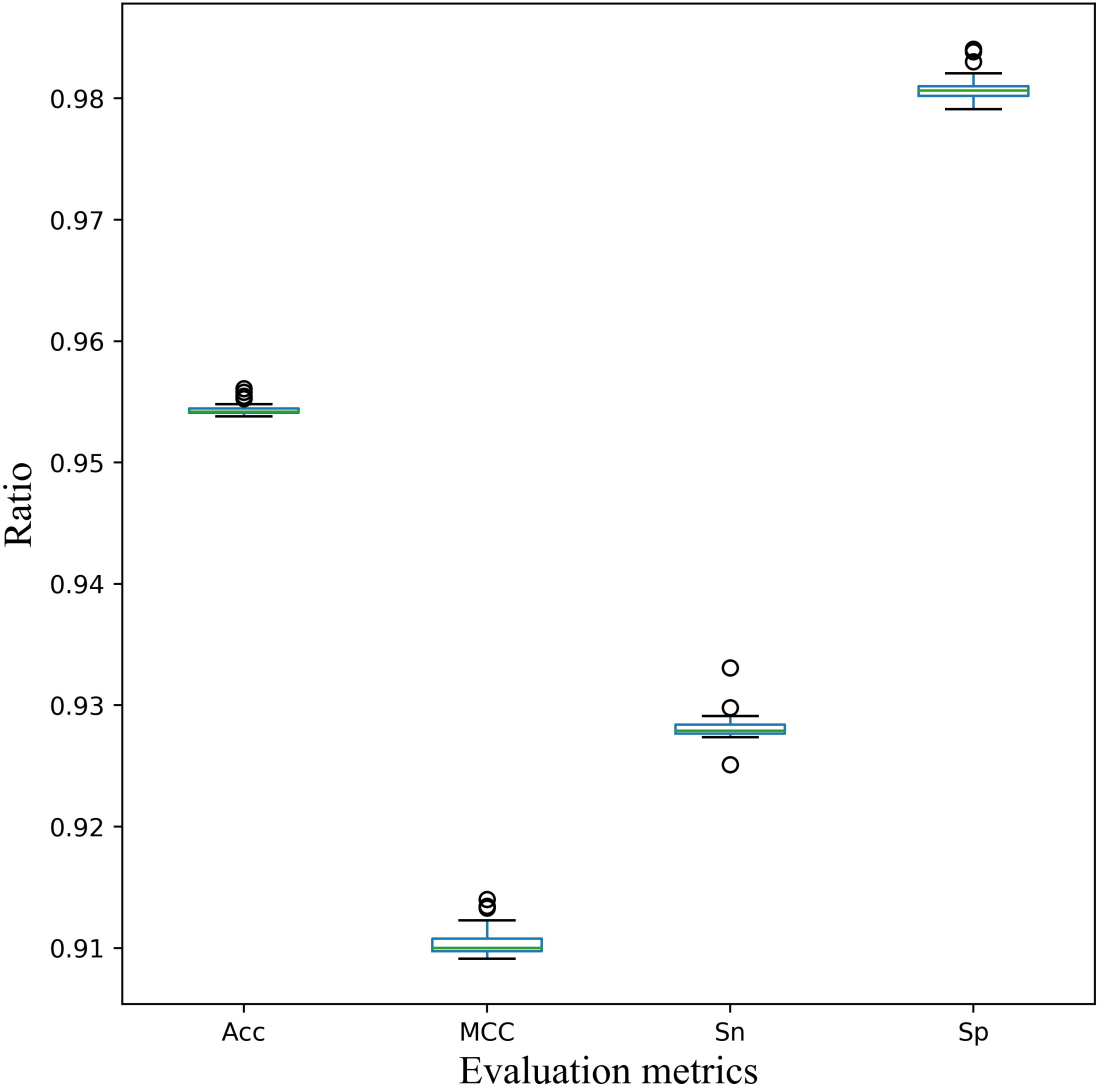
Stability of the EnACP model on 5-fold cross-validation dataset. Five-fold cross validation experiment was executed 30 times randomly. And the metrics of Acc, MCC, Sn and Sp were plotted and analyzed.

Furthermore, for the stability of the model, 5-fold cross validation experiment was executed 30 times randomly. According to the statistical results of various evaluation metrics shown in Figure 5, several indicators fluctuate little. And the standard deviation of Acc, MCC, Sn, and Sp is 0.0005, 0.0012,0.0012, and 0.0011, respectively. Therefore, the cross-validation analysis showed the stability and robustness of our model EnACP.

### Performance Comparison on Independent Test Datasets

To further verify the power of the current predictor, three independent datasets are analyzed from mACPpred (Boopathi et al., 2019), ACPP (Vijayakumar and Lakshmi, 2015), and Tyagi's paper (Tyagi et al., 2013) named mACP_Ind, ACPP_Ind, and Tyagi_Ind, respectively. For the independent test dataset mACPpred_Ind, SVMACP and RFACP belong to MLACP algorithm based on RF and SVM method, respectively. For this dataset, we refer to the experimental results from the literature mACPpred (Table 2). And for the independent test datasets ACPP_Ind and Tyagi_Ind, we compare our algorithms EnACP with mACPpred and iACP (Table 3). Experimental results on independent tests show that this proposed EnACP predictor is quite more effective and promising for identification of ACPs compared with the previous methods.

**Table 2:** Performance comparison of different methods on the independent test dataset mACPpred_Ind.

**Methods**	**Acc**	**Sn**	**Sp**	**MCC**	**AUC**
EnACP	0.924	0.892	0.955	0.849	0.968
mACPpred	0.914	0.885	0.943	0.829	0.967
SVMACP	0.768	0.554	0.981	0.592	0.896
RFACP	0.707	0.414	1.000	0.511	0.891
iACP	0.667	0.580	0.753	0.338	0.747

**Table 3:** Performance comparison of different methods on the independent test datasets ACPP_Ind and Tyagi_Ind.

**Datasets**	**Methods**	**Acc**	**Sn**	**Sp**	**MCC**	**AUC**
	EnACP	0.948	1	0.9	0.901	0.992
ACPP_Ind	mACPpred	0.948	0.973	0.925	0.898	0.989
	iACP	0.74	0.919	0.575	0.558	0.875
	EnACP	0.853	1	0.708	0.739	0.996
Tyagi_Ind	mACPpred	0.884	0.957	0.813	0.777	0.948
	iACP	0.8	0.894	0.708	0.612	0.905

Compared with mACPpred method, our model EnACP had achieved excellent results, among which, MCC, Acc, Sn, and Sp were all about 2, 1, 0.7, and 1.2% higher, respectively, AUC was basically flat. MCC, Acc, Sn, and AUC obtained from our model EnACP were about 25.7 to 51.1%, 15.6 to 25.7%, 31.2 to 47.8%, 7.2 to 22.1% higher, respectively, compared with SVMACP, RFACP, and iACP. Additionally, it can also be seen from the results of Figure 4 and Table 2 that the EnACP method has an advantage over the pseudo amino acid composition method with one step prediction. Sn is only slightly lower less than a percentage point. And, MCC, Sp, Acc, and AUC obtained from EnACP model were about 4, 4, 2, 2% higher than the pseudo amino acid composition method with one step prediction. For ACPP_Ind and Tyagi_Ind datasets, EnACP achieves the similar performance advantages on AUC and Sn.

The statistical significance is evaluated using rank-based ROC curves comparison to determine whether EnACP performs better than, similarly to or worse than the other algorithms (DeLong et al., 1988; Hanley and Hajian-Tilaki, 1997). The results are shown in the following Table 4. For a confidence level of 0.95, EnACP perform statistically significantly better than iACP on all datasets. EnACP performs similarly or slightly better than mACPperd algorithms on mACP_Ind and ACPP_Ind. And mACPpred performs better than iACP on the previous two datasets. The algorithms EnACP and mACPperd perform better than iACP with statistical significance. The comparison triplets are also statistically tabulated between algorithm pairs from EnACP, mACPpred and iACP which show that one algorithm performs better, equally well and worse, compared with another algorithm in Table 5.

**Table 4:** Pairwise comparison of ROC curves in three datasets.

**Datasets**	**P(A, B)**	**EnACP**	**mACPpred**	**iACP**
mACP_Ind	EnACP	—	0.9705	<0.0001
	mACPpred	—	—	<0.0001
	iACP	—	—	—
ACPP_Ind	EnACP	—	0.6612	0.0036
	mACPpred	—	—	0.0076
	iACP	—	—	—
Tyagi_Ind	EnACP	—	0.0384	0.0015
	mACPpred	—	—	0.2381
	iACP	—	—	—

*The comparison P(A, B) is defined the statistical significance P-value of ROC curves between algorithm A and algorithm B*.

**Table 5:** The comparison triplets between algorithm pairs from EnACP, mACPpred and iACP.

**T(A,B)**	**EnACP**	**mACPpred**	**iACP**
EnACP	—	1/2/0	3/0/0
mACPpred	0/2/1	—	2/1/0
iACP	0/0/3	0/1/2	—

*The comparison triplet T(A, B) is defined to be the numbers of the three datasets where algorithm A performs better, equally well and worse, compared with algorithm B in terms of P-value*.

### Comparison of Different Classification Methods

Based on many previous studies, using SVM classifier for task of peptide classification outperforms most of other classical classifiers such as AdaBoost, decision tree (DT), logistic regression (LR), Naïve Bayes (NB), random forest (RF) (Becker et al., 2011). We also conducted a comparative study on the two datasets and obtained the similar conclusion in the second step of the model EnACP. Experimental results on both the 5-fold cross-validation and independent test showed that SVM, NB and LR were relatively stable, and SVM has the best overall effect.

**Figure 6:**
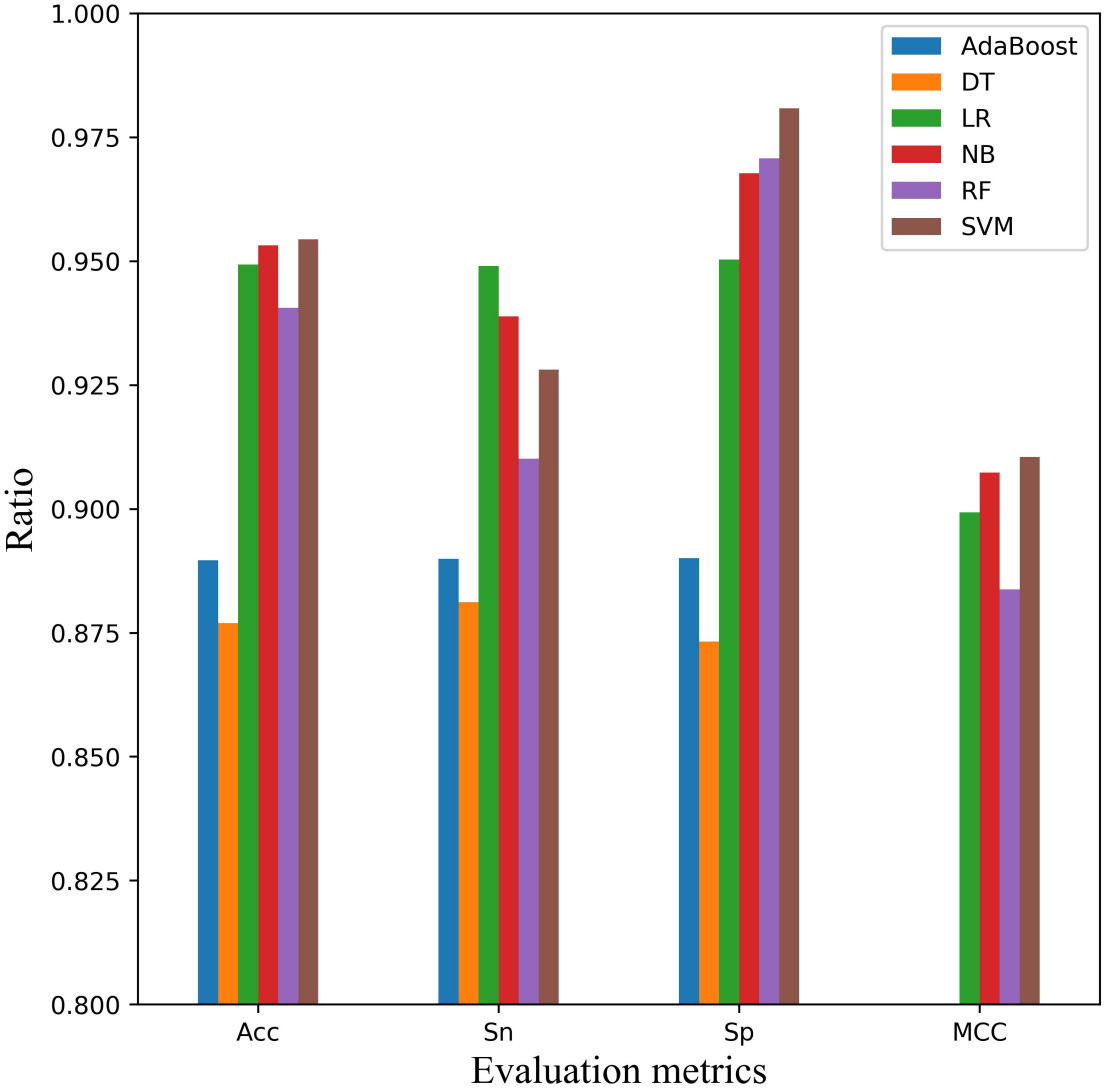
Comparison of SVM with other classifiers on 5-fold cross-validation dataset. Four performance indicators which are Sn, Sp, Acc, and MCC are compared using six classifiers that are AdaBoost, decision tree (DT), logistic regression (LR), Naïve Bayes (NB), random forest (RF), and Support Vector Machine (SVM), respectively.

In order to verify the performance of SVM classifier, we randomly selected scrambled data before 5-fold cross-validation. Finally, the average result of six classifiers were obtained after 30 times of 5-fold cross validation, as shown in Figure 6. Each classifier performed well, but in the comprehensive comparison, SVM, LR, and NB classifiers were better. On the whole, SVM classifier worked best. SVM achieved the first place in the three indexes of Acc, MCC, and Sp. For the Sn index, it was only about 1 and 2% lower than the classifier of NB and LR, respectively.

**Figure 7:**
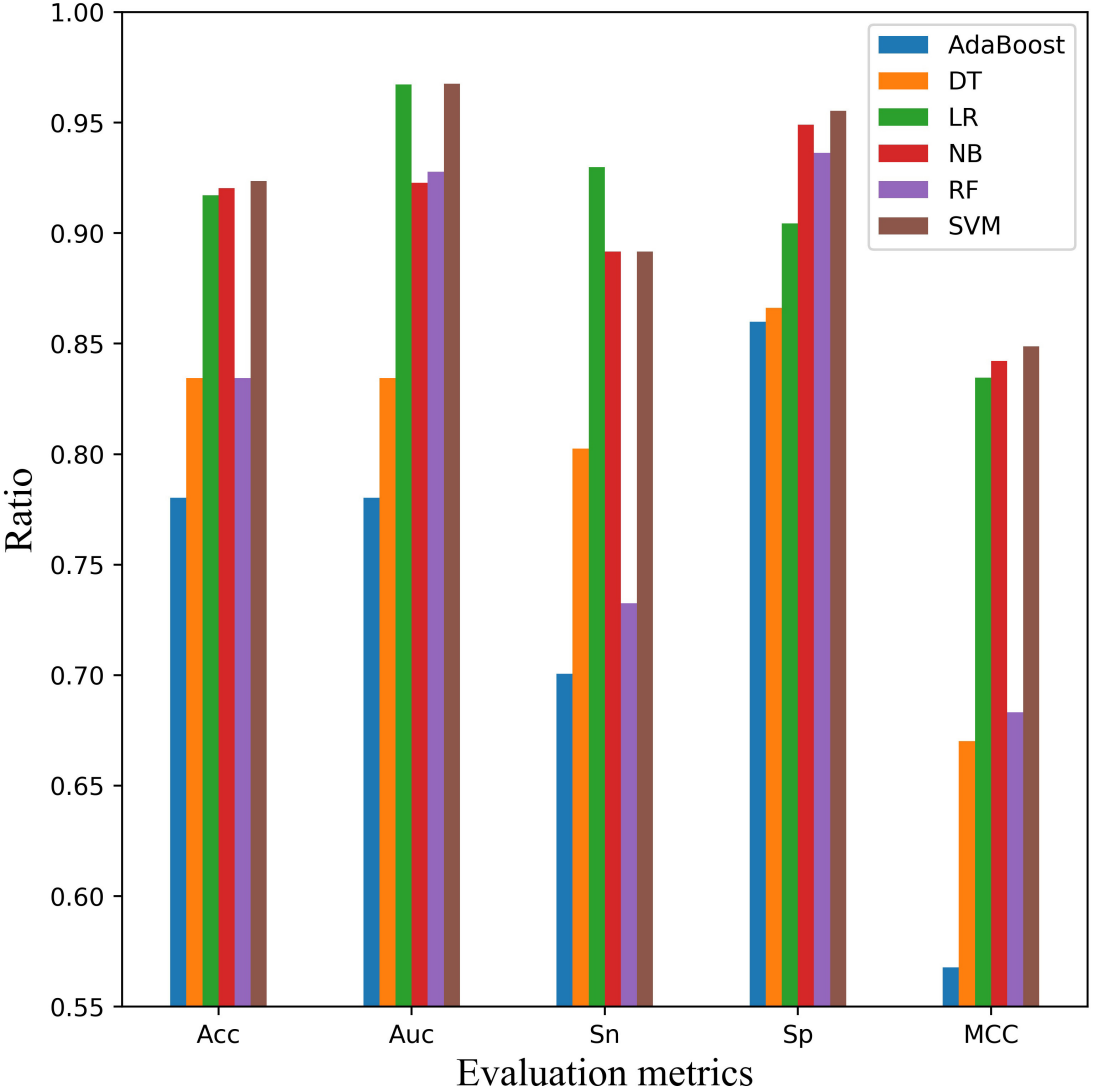
Comparison of SVM with other classifiers on independent test dataset mACPpred_Ind. Five performance indicators which are AUC, Sn, Sp, Acc, and the Matthews correlation coefficient (MCC) are compared using six classifiers that are AdaBoost, decision tree (DT), logistic regression (LR), Naïve Bayes (NB), random forest (RF), and Support Vector Machine (SVM), respectively.

In addition, independent test dataset mACPpred_Ind was used to measure the performance and categorization capabilities of the optimal model in Figure 7. Compared with the cross-validation experiment, the AUC evaluation metric was added into this experiment except Acc, Sn, Sp, and MCC. Except for Sn, SVM classifier ranked the first place in Acc, AUC, Sp, and MCC, which was similar to the cross-validation result. But, SVM had better performance relative to cross validation tests. For example, for AUC index, SVM was more than 13 points higher than AdaBoost and DT. For Sp index, SVM is more than 5 points higher than AdaBoost, LR and DT. For MCC, SVM was 16% higher than RF and DT.

## Discussion

Even to this day, it is difficult to trace the cause of cancer because of its complex mechanisms. In spite of various treatment strategies, the effect was not ideal. Peptide-based therapy has become a research field of precision medicine. The rapid and accurate identification of ACPs from peptide sequences based on machine learning methods can be better applied to anticancer drug development and other biomedical experiments (Diller et al., 2018).

From the experimental results of the independent test datasets, our model EnACP performs well overall especially the high AUC and sensitivity. The higher the sensitivity is, the better the predicted model of ACPs is. The highly sensitive discovery of anticancer peptides plays an important role in the design of anticancer and anti-tumor synthetic drugs. The innovation of our model mainly includes the following points. The model EnACP is robust and easy to extend. Multi-group feature encodings contain abundant information. For each group of feature encoding, LightGBM as the first layer of EnACP can auto pre-learning and select the key features, respectively. Actually, for the higher-dimensional features, the computation is not very large. Meanwhile, the model implements the multi-layer feature learning strategy. Moreover, the second layer has fewer features and the model is more efficient to identify the ACPs and non-ACPs. The proposed EnACP performs better in identifying whether the peptide sequence is ACP compared with the existing methods. Its accuracy and stability may be attributed to the following reasons.

At first, how to effectively extract the valuable information of ACPs is a major challenge for all the predicted methods. It has been proved that the membrane interaction and insertion of membrane-active peptides could be related to the order of amino acids. Systematic analysis revealed that some physiochemical properties of peptides are not clearly sufficient to predict their selectivity for example net positive charge, hydrophobicity, and hydrophobic moments (Chen W. et al., 2016). Some methods also are developed using amino acid composition and binary profiles as input features (Lin et al., 2015). Therefore, in order to find a suitable feature representation, EnACP extracts 19 kinds of features from four aspects, including amino acid composition, auto correlation, pseudo amino acid composition and profile-based features.

Then, in purpose to accurately identify the ACPs quickly, LightGBM classifier is applied to detect the peptide sequences with the 19 kinds of features. As an ensemble learning method, LightGBM can automatically optimize to achieve dimension reduction and effectively prevent overfitting. On the other hand, it can better discover the relationship of peptides and select the representative feature description from the integrating multiple groups features (Huang et al., 2010). In addition, the secondary structure and tertiary structure prediction characteristics of peptides can be added into this model as a part of basis feature description, which may further improve the performance of the model (Ma et al., 2015). Furthermore, neural network method can also be explored for the identification of ACPs with the increase of datasets (Hashemifar et al., 2018).

Finally, in terms of the used classifiers, many prediction tools have demonstrated the effectiveness of the SVM method. As a two-step prediction model, SVM finally outputs the identified results with grid search to optimize its parameters. Besides, in order to expedite the identification of ACPs, we called LightGBM with the default parameters in the scikit-learn package library. Better model parameters may be obtained by modern optimization methods to improve the prediction performance.

## Conclusion

In order to effectively identify ACPs from amino acid sequences, a novel hybrid predicted model EnACP is proposed in this paper. EnACP involves two-step strategy based on ensemble learning method. Firstly, multi-type and multi-group feature descriptions were constructed based on amino acid composition, autocorrelation, pseudo amino acid composition and profile-based features. In purpose to find a suitable feature representation and accurately classify quickly, the ensemble classifier LightGBM was applied to detect the peptide sequences. Secondly, multiple groups of results from the output of LightGBMs were integrated as the input of SVM model to enhance the final prediction accuracy of ACP as well as non-ACP. To validate the performance of EnACP, two group experiments were performed on cross validate dataset and independent dataset. The experimental results indicated that the proposed EnACP model achieved competitive performance on some performance metrics. On the other hand, our model can be used to solve other protein sequence problems, such as homologous detection of proteins (Chen J. et al., 2016), prediction of various sites (Chou and Shen, 2008, 2010), prediction of protein-protein interaction (Wang et al., 2019), etc.

## Data Availability Statement

Publicly available datasets were analyzed in this study. The datasets can be found in: https://github.com/greyspring/EnACP/tree/master/datasets.

## Author Contributions

RG and PW designed the method. RG and GF developed the prediction models. GF, XJ, RZ, and QW analyzed the data and results. All authors have read and approved the revised manuscript.

## Conflict of Interest

The authors declare that the research was conducted in the absence of any commercial or financial relationships that could be construed as a potential conflict of interest.
